# Surface Crack Detection of Aluminum Alloy Using Injected Direct Current-Magnetic Field Measurement Method

**DOI:** 10.3390/s25061800

**Published:** 2025-03-14

**Authors:** Huipeng Wang, Jialong Shi, Qiaogen Wang, Lihong Dong, Huizhong Liu

**Affiliations:** 1 School of Mechanical and Electrical Engineering, Jiangxi University of Science and Technology, Ganzhou 341000, China; wanghuipeng1983@126.com (H.W.); 6720220845@mail.jxust.edu.cn (J.S.); 15707963656@163.com (Q.W.); huizhong6@163.com (H.L.); 2 National Key Laboratory for Remanufacturing, Army Academy of Armored Forces, Beijing 100072, China

**Keywords:** aluminum alloy, injected direct current-magnetic field measurement, peak-to-peak value Δ*B_xpp_*, crack width, crack depth

## Abstract

Injected Direct Current-Magnetic Field Measurement (IDC-MFM) is a promising nondestructive technique for crack evaluation of aluminum alloy due to its high sensitivity to defect states. Finite element simulation and experiment were introduced in this research to reveal the relationship between the magnetic field and the crack size. The finite element simulation showed that the magnetic field at the defect increased with the currents, and the peak-to-peak value of the tangential component magnetic field *B_x_* (Δ*B_xpp_*) correlated with both the width and depth of the crack very well. The high-sensitivity tunnel magnetoresistance probe was used for crack detection of aluminum alloy specimens with different crack widths and depths, and the results show that the relationship between the Δ*B_xpp_* and the crack was consistent with the simulation results, and Δ*B_xpp_* has a nonlinear positive correlation with crack width and a linear positive correlation with crack depth. The results of the present work show that IDC-MFM has high sensitivity in crack size detection and is a feasible solution for the quantitative evaluation of cracks in aluminum alloy.

## 1. Introduction

Aluminum alloys are widely used in the aerospace, automotive and construction industries due to their high strength, corrosion resistance and excellent processing properties [1,2]. However, aluminum alloy components are prone to cracks and other defects during processing and service [3], which significantly affects their mechanical properties and even leads to component fracture and failure. Width and depth are important dimensions of the crack [4]. The crack width reflects the degree of opening, which affects the stress distribution of the materials and the invasion rate of external media. The crack depth determines the degree of damage to the internal structure of the material, which significantly affects the load-bearing capacity of the material. Therefore, accurate crack size evaluation is essential to ensure the service safety of materials [5,6].

Injected Direct Current-Magnetic Field Measurement (IDC-MFM) was developed based on DC Potential Drop (DCPD) and Magnetic Flux Leakage (MFL) technology [7]. It is an electromagnetic nondestructive testing method suitable for metal materials, especially non-ferromagnetic metal materials [8]. IDC-MFM uses magnetic sensors to measure the abnormal magnetic field caused by cracks. Since the direct current is introduced into the specimen, it is not affected by the skin effect [9], so it can effectively detect the internal defects of the specimen and is sensitive to crack depth detection.

Recently, IDC-MFM has great application potential in defect detection and has achieved many meaningful results. In the feasibility study [10,11], the change in magnetic field caused by current disturbances at the defect is used as the theoretical basis for detection. When detecting electronic circuits [12,13], the magnetic field generated by the short-circuited chip stack was mapped to enable fault location evaluation. Wikswo et al. [14] used the SQUID magnetometer (Vanderbilt University, Nashville, US, USA) to detect through-hole defects in thin copper plates. Sebestyen et al. [15] used the finite element method to study the feasibility of detecting complex structural defects and identifying internal defects. Inoue et al. [16] detected the internal defects of coated conductors and investigated the influence of the lift-off value on the detection technology. Hafiz et al. [17] used wavelet analysis to determine the crack position of the conductive plate, and the results can enhance the multiple crack detection ability. Li et al. [18,19] used IDC-MFM to detect surface cracks in aluminum alloys, which further confirmed the wide applicability of this technology in defect detection. However, in the current research, the magnetic signal properties for surface crack detection of aluminum alloys are not yet clearly defined and the IDC-MFM is able to detect defects qualitatively and quantitatively based on magnetic field characteristics. Therefore, the use of this technology for surface crack detection of aluminum alloys should be further investigated.

The IDC-MFM has made great progress in defect detection; however, quantitative analysis of crack width and depth in aluminum alloy is lacking. Therefore, an aluminum alloy model with a prefabricated crack was developed to investigate the feasibility of IDC-MFM technology for quantitative crack detection on aluminum alloy surfaces in this simulation work. In combination with experimental verification, the effects of excitation intensity, crack width and depth on magnetic field were discussed. The results are expected to provide theoretical foundations for the application of IDC-MFM in crack detection of aluminum alloys.

## 2. Theories of IDC-MFM

The principle of IDC-MFM is based on Maxwell equations. When direct current is injected into uniform plates, a magnetic field change occurs in the adjacent space. The calculation of the surface magnetic field can be expressed by the Biot-Savart Law as [20]

(1) $$dB=\frac{\mu_{0}}{4\pi }\frac{Idl\times \hat{r}}{r^{2}}$$

where $\mu_{0}$ is the vacuum permeability, Idl is the current element, $\hat{r}$ is the unit vector from the source point to the field point and r is the distance from the source point to the field point.

The magnetic field of the complex defects is calculated by the influence of the current density at the source point on the field point, which can be expressed as

(2) $$\vec{\nabla }\times \vec{B}=\mu_{0}\vec{J}$$

where $\vec{B}$ is the magnetic fields, $\vec{\nabla }$ is the curl of the field point and $\vec{J}$ is the current density.

The tangential component of the magnetic field, *B_x_* signal, is the quantity of interest because it can be measured by the current test equipment. The current density component that can generate *B_x_* is in the *y* and *z* directions when the conductor is injected with direct current in the *x* direction. For a good conductor, the relationship between the current density J and the electric field *E* is J=σE, where σ is the conductivity. Then, the current density component can be expressed as [21]

(3) $$J_{x}=\sigma_{xx}E_{x}+\sigma_{xy}E_{y}+\sigma_{xz}E_{z}$$

(4) $$J_{y}=\sigma_{yx}E_{x}+\sigma_{yy}E_{y}+\sigma_{yz}E_{z}$$

(5) $$J_{z}=\sigma_{zx}E_{x}+\sigma_{zy}E_{y}+\sigma_{zz}E_{z}$$

Thus, the *B_x_* of the specimen is expressed by the Biot-Savart Law as follows:

(6) $$B_{x}=\frac{\mu_{0}}{4\pi }\sum_{i}\int_{\Delta vi}J_{i}\frac{\left(R-r\right)}{{\left|R-r\right|}^{3}}dv\left(r\right)$$

where *B_x_* is the magnetic field at the field point $R=\left(x,y,z\right)$, $J_{i}$ is the value of the current density in the *y* and *z* directions, $\Delta v_{i}$ is the finite volume when the current density $J_{i}$ and the source point r are determined and $\mu_{0}$ is the vacuum permeability.

**Figure 1:**
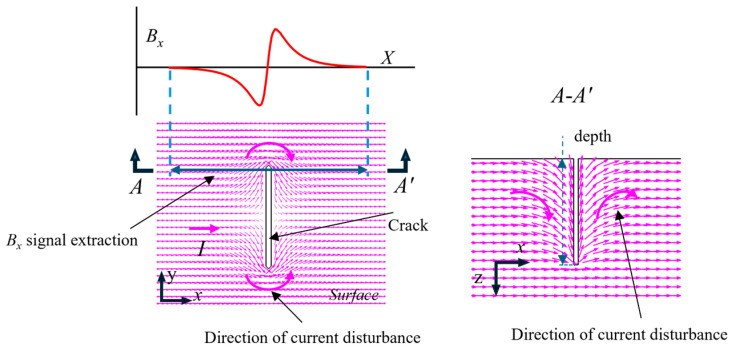
Schematic of the current density along the surface and the depth direction.

Figure 1 is the current density vector diagram of the plane and depth direction of the crack specimen. When there is a defect, the parallel current flows along the defect boundary, and many *J_y_* and *J_z_* current density components are generated at the crack boundary. These components are closely related to the width or depth of the crack. The current density component caused by the defect generates a *B_x_* magnetic field, and the *B_x_* changes from zero to a new signal, indicating the magnetic field associated with the defect. The current circular trend results in opposite magnetic fields peaking on the left and right sides of the crack, and there are opposite signal phenomena in the two peak directions of the crack. According to the above analysis, the *B_x_* signal can be used for width and depth detection of the crack.

## 3. Finite Element Simulation

### 3.1. 3D Modelling

**Figure 2:**
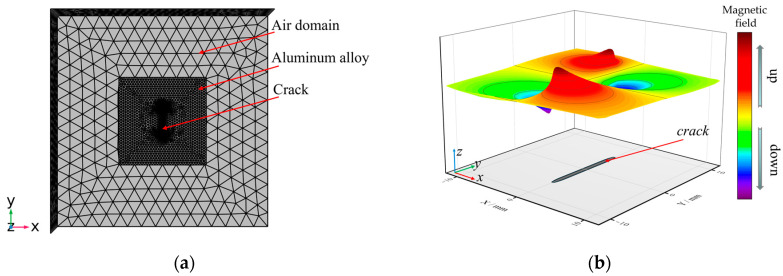
3D modelling analysis: (**a**) 3D model; (**b**) normalized surface magnetic field.

A 3D model of the electromagnetic field finite element model of aluminum alloy specimens with cracks has been developed in COMSOL Multiphysics 6.0, as shown in Figure 2a. The specimen was 7075 aluminum alloy, the conductivity was set to 1.9 × 10^7^ S/m and the relative permeability was set to 1.00022. The geometric size of the specimen was 40 mm × 40 mm × 5 mm (length × width × thickness). The prefabricated surface cracks were rectangular grooves. The length, width and depth parameters were set for subsequent research. The air domain surrounds the specimen, the size was 100 mm × 100 mm × 50 mm and the relative permeability was set to 1.0. The mesh was set as a free tetrahedral element, and the mesh refinement was applied to the crack area. The minimum element size was 0.1 mm, the maximum element growth rate was 1.5 and the number of calculated mesh elements was 3.1 × 10^5^. Similarly, in another mesh distribution set, the number of mesh elements was doubled. Through mesh convergence analysis, the difference in the surface magnetic field calculation results of the two was less than 1%, which indicated that the current mesh distribution was capable of simulation.

The current excitation was direct current (DC), and the current was injected along the *x*-axis of the specimen. Magnetic field detection was set at the upper surface of the specimen, with a distance of 0.05 mm between the measurement points. The boundary conditions were as follows: magnetic insulation conditions were applied at the outer boundary of the air domain (*n* × A = 0), and continuous boundary conditions were applied at the interface between the specimen and the air. For the boundary between conductor regions, the normal component of the current density is continuous, and meet the following boundary conditions:

(7) $$n\cdot \left(J_{1}-J_{2}\right)=0$$

where $J_{1}$ and $J_{2}$ are the normal components of the current density of the boundary, respectively.

### 3.2. Simulation

In the simulation analysis, considering the abnormal current density phenomenon caused by defects, the coupling calculation of electric field and magnetic field was adopted. Through geometric analysis, the current density distribution in the model was calculated using COMSOL electric field analysis. Meanwhile, the magnetic field distribution of the model was calculated using the steady-state magnetic field analysis, and the vector intensity of the magnetic field was calculated using the current density analysis results. In steady-state simulation, the direct current equation is ∇⋅J=0. The analysis of the magnetic field generated by the constant current is shown in Equation (2).

The relative permeability of aluminum alloy is 1.00022, and the relative permeability of the surrounding air and the air within the defect is 1. Considering that the magnetic induction intensity of aluminum alloy specimen and defects under the action of geomagnetic field is relatively weak [22], the influence of geomagnetic field has not been studied. After the solution was completed, the magnetic field distribution cloud was extracted from the post-processing program. The direction of the tangential component of the magnetic field was along the *x*-axis, as shown in Figure 2a. The normalized surface magnetic field intensity was shown in Figure 2b, and there was a peak-to-peak characteristic above the crack area, and it is the strongest at the crack tip. The peak-to-peak value of *B_x_* can be expressed as

(8) $$\Delta B_{xpp}=B_{xmax}-B_{xmin}$$

where Δ*B_xpp_* is the peak-to-peak value of *B_x_*, *B_xmax_* is the maximum of *B_x_* and *B_xmin_* is the minimum of *B_x_*.

### 3.3. Simulation Results

#### 3.3.1. Influence of DC Intensity on Δ*B_xpp_*

The application of direct current (DC) as the excitation source created a stable magnetic field in the detection object and significantly increased the abnormal magnetic field at the defect. There was a significant magnetic field difference between the defect and its surrounding normal area, which provided better conditions for defect detection.

**Figure 3:**
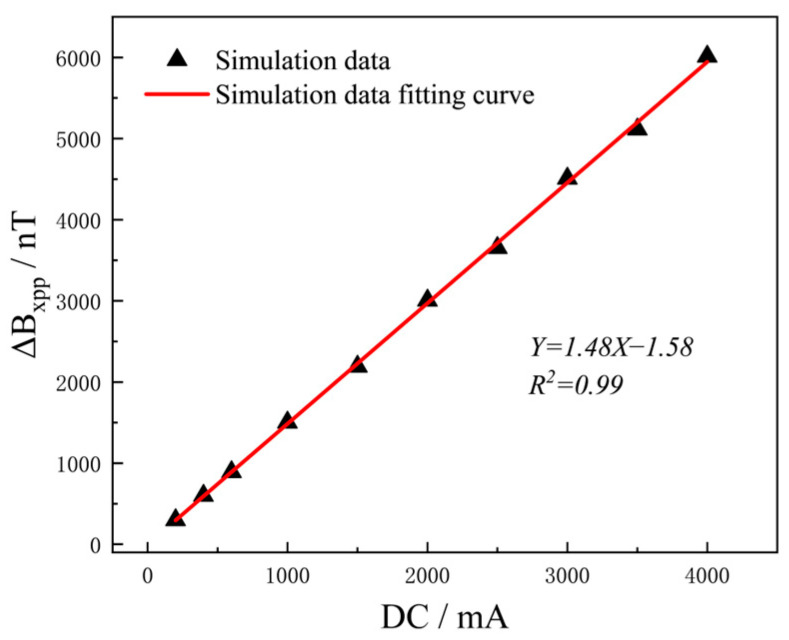
Variation of Δ*B_xpp_* with the DC intensity.

In this simulation, aluminum alloy specimens with single defects (length, width and depth: 12 mm × 0.5 mm × 2.5 mm) were tested with different current intensities from 200 mA to 4000 mA, and Δ*B_xpp_* was selected for research and analysis. As shown in Figure 3, as the current increased, Δ*B_xpp_* also increased linearly. This indicates that the effect of current excitation on the abnormal magnetic field generated at the crack was unsaturated within a certain range. It can be seen that by adjusting the excitation current intensity, multi-size defect detection can be realized. Increasing the excitation intensity directly affects the magnetic field at the defect, thereby improving the detection ability of the defect. However, currents that are too high can lead to overload damage to the test object. Therefore, detection should consider factors such as the carrying current capacity of the detection object, the measurement range and the resolution of the detection equipment to determine the current application intensity.

From the above research, it can be seen that the amplitude of the crack signal under current excitation of 1500 mA meets the sensitivity detection requirements of high-sensitivity magnetic sensors, so 1500 mA was selected as the excitation current intensity for subsequent research.

#### 3.3.2. Influence of Crack Width on Δ*B_xpp_*

**Figure 4:**
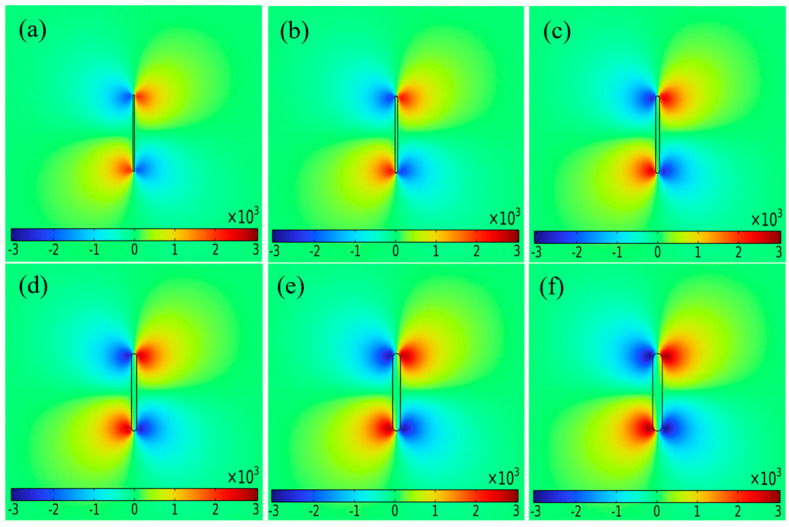
Magnetic field cloud under different crack widths with DC intensity of 1500 mA: crack width (**a**) 0.2 mm; (**b**) 0.4 mm; (**c**) 0.6 mm; (**d**) 0.8 mm; (**e**) 1.0 mm; (**f**) 1.2 mm.

Figure 4 shows the distribution of magnetic fields on the surface of the aluminum alloy specimen with cracks of different widths under DC intensity of 1500 mA. The cracks were 12 mm long and 2.5 mm deep, with crack widths of 0.2, 0.4, 0.6, 0.8, 1.0 and 1.2 mm, respectively. The magnetic field cloud showed that the crack width was positively correlated with the magnetic field, and the magnetic field at the crack was significantly different from the surrounding area. Even a tiny crack with a width of 0.2 mm caused significant changes in the magnetic field. In the current disturbance effect, the current flows along the edges of the defect, and the abnormal current at the crack tip was more pronounced than that at the center of the crack. As the crack width increased, the current deflection became more noticeable and the magnetic field at the crack tip increased.

**Figure 5:**
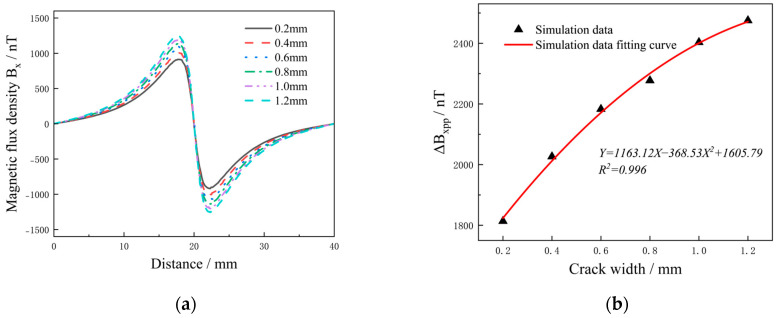
*B_x_* under different crack widths: (**a**) Surface magnetic field; (**b**) Δ*B_xpp_* with crack widths.

To analyze the relationship between surface magnetic field and crack width of the aluminum alloy specimen, a signal acquisition path extending 20 mm to the left and right was established at the crack tip, with the defect center serving as a reference point. The data were shown in Figure 5a. The results showed that the signal at the crack had a peak-to-peak magnetic field, with both the amplitude of the magnetic field curve and the area of abnormal signal increasing as the crack width increased.

Δ*B_xpp_* of six defect signals were extracted, and the binomial function was fitted according to the crack width. As shown in Figure 5b, the fitted plot of Δ*B_xpp_* with crack width was nonlinear. The functional formula obtained from the simulation data is *Y* = 1163.12*X* − 368.53 × 2 + 1605.79, and can be used to predict the defect width. Δ*B_xpp_* increased rapidly and then tended to slow down, suggesting that there was a relatively sensitive interval in crack width detection. However, under the condition of a certain crack depth, cracks with different widths showed high signal gradient values in the detection results.

#### 3.3.3. Influence of Crack Depth on Δ*B_xpp_*

**Figure 6:**
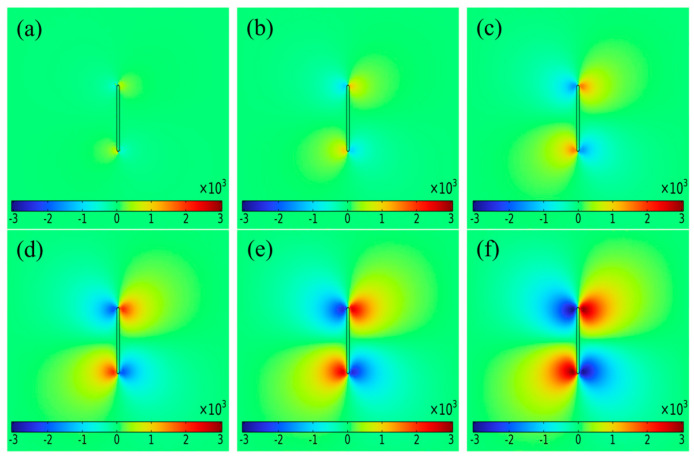
Magnetic field cloud under different crack depths with DC intensity of 1500 mA: crack depth (**a**) 0.5 mm; (**b**) 1.0 mm; (**c**) 1.5 mm; (**d**) 2.0 mm; (**e**) 2.5 mm; (**f**) 3.0 mm.

Figure 6 shows the distribution of magnetic fields on the surface of aluminum alloy specimens with cracks of different depths under current intensity of 1500 mA. The cracks were 12 mm long and 0.5 mm wide, and the crack depths were 0.5, 1.0, 1.5, 2.0, 2.5 and 3.0 mm, respectively. The magnetic field cloud showed a positive correlation between crack depth and magnetic field. However, at shallower crack depths (0.5 mm), the abnormal magnetic field behaved relatively weakly.

**Figure 7:**
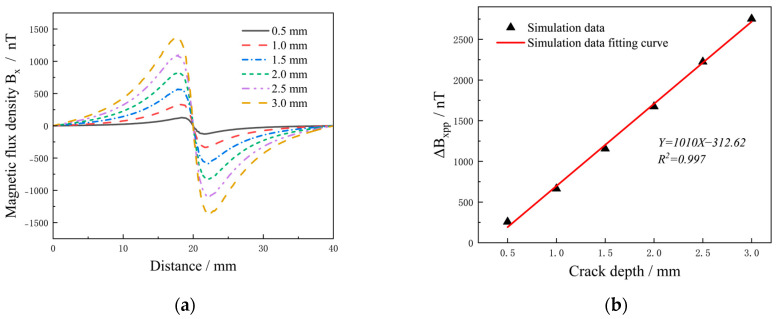
*B_x_* under different crack depths: (**a**) surface magnetic field; (**b**) Δ*B_xpp_* with crack depth.

As shown in Figure 7a, *B_x_* increased significantly with increasing crack depth. Δ*B_xpp_* of the six defects were extracted and adjusted according to the crack depth. As shown in Figure 7b, there was a linear positive correlation between Δ*B_xpp_* and crack depth. The functional formula obtained from the simulation data was *Y* = 1010*X* − 312.62, and could be used to predict the defect depth. It can be seen from Figure 7a,b that when the crack depth was too small, the *B_x_* was relatively weak and the region of the magnetic field area caused by the defect decreased significantly. As the crack depth increased, Δ*B_xpp_* increased significantly, indicating that the crack depth was a crucial factor affecting the magnetic field of the defect.

## 4. Experimental

### 4.1. Experimental Setup

#### 4.1.1. Specimens

**Figure 8:**
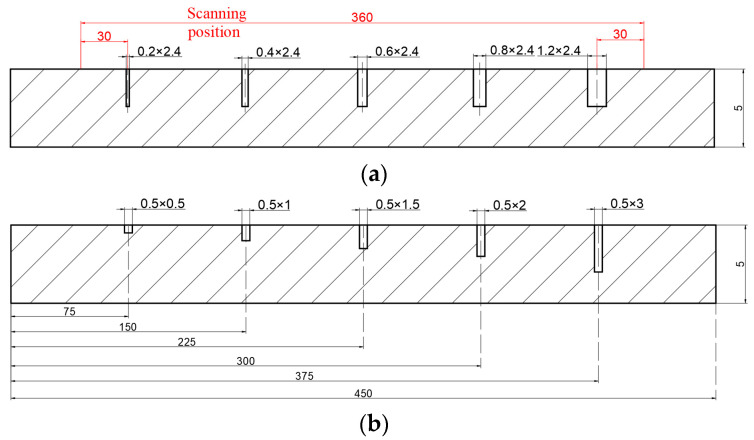
Cross-section schematics of the experimental specimens with different widths (**a**) and depths (**b**).

The specimen material was a high-strength 7075 aluminum alloy, the chemical composition and physical properties of which are as shown in Table 1 and Table 2. To investigate the influence of factors such as crack width and depth, two groups of different types of defect specimens were fabricated. The aluminum alloy plate was machined with a CNC lathe and had a size of 450 mm × 40 mm × 5 mm. Five cracks with the same finite element simulation were machined into the surface of the specimen, each crack length being 12 mm, as shown in Figure 8.

#### 4.1.2. Instruments

**Figure 9:**
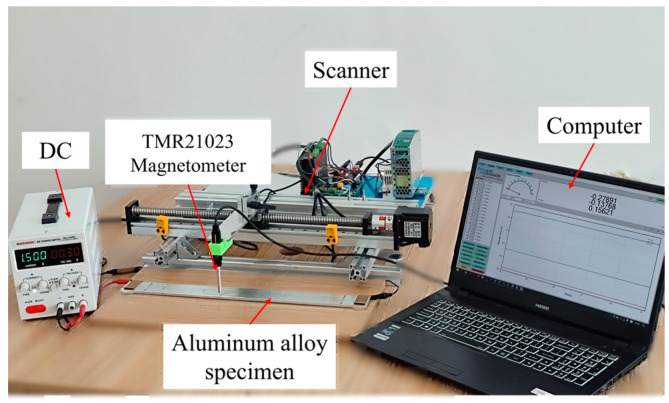
Experimental equipment setup.

The TMR21023 magnetometer (MultiDimension Technology Co., Suzhou, China) with a measuring range of ±10 mT, a resolution of 1 nT and an RMS noise of 20 nT was used for signal acquisition. The DC excitation equipment was MS-155DS (MAISHENG, Shenzhen, China) with a resolution of 1 mA. The smallest controllable step for controlling the movement system of the magnetometer was 0.03 mm. The test platform constructed with the above equipment was used to test the specimen. The test system is arranged as shown in Figure 9.

#### 4.1.3. Experimental Arrangement

The tests were carried out in the laboratory, and the specimen was far away from other ferromagnetic materials. The probe was connected to the upper computer, the lift-off value of the probe was set to 1 mm and the scan path was selected at the crack tip with a length of 360 mm, as shown in Figure 8a. The scanning speed was set at 5 mm/s with the probe synchronized with the scanning platform. Then, the DC excitation detection was carried out, and the collected signal of the defect was subjected to magnetic field compensation with the environmental magnetic field and background noise [23]. Mathematical operation of background magnetic field compensation is

(9) $$B=B_{meas}-B_{b}$$

where *B_meas_* is the original measured magnetic signal, *B_b_* is the background magnetic field and *B* is the magnetic signal after background compensation.

## 5. Results

### 5.1. Detection Results of Different Current Intensities

**Figure 10:**
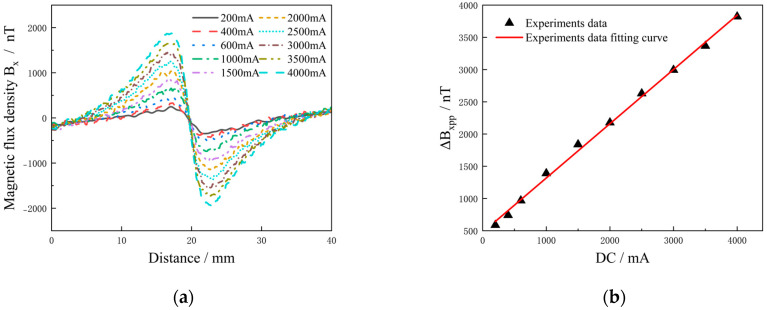
*B_x_* under different DC intensities: (**a**) surface magnetic field; (**b**) Δ*B_xpp_* with DC intensities.

In this experiment, the same current excitation (200−4000 mA) as in the finite element simulation was used to detect the aluminum alloy plate with a single defect (length, width and depth: 12 mm × 0.5 mm × 2.5 mm). As shown in Figure 10a, the *B_x_* increased with increasing current intensity. As shown in Figure 10b, the excitation intensity was linear to Δ*B_xpp_*, which is consistent with the simulation results in Figure 3. IDC-MFM is based on the principle that the steady-state current induces a magnetic field, and is in accordance with the principle of magnetic superposition. The magnetic field generated by each current at the defect is equal to the sum of the vectors of all the current elements at the defect, that is, $B=\sum_{i}B_{i}$. It can be verified that magnetic field intensity and current excitation show a linear monotonic relationship, and the simulation results are consistent with the experimental results. In this experiment, the detected environmental magnetic field fluctuation was ± 10 nT and the magnetic field resolution of the detection equipment was 1 nT. The signal amplitude at the defect went as high as 1840 nT when the excitation intensity was 1500 mA.

### 5.2. Detection Results of Cracks with Different Widths and Depths

**Figure 11:**
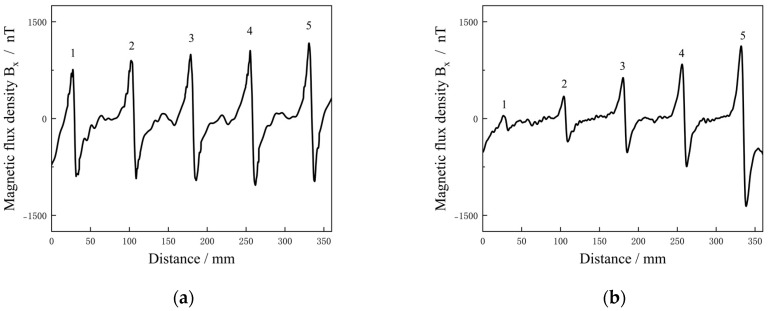
*B_x_* of the specimens with different crack widths (**a**) and depths (**b**).

Two groups of specimens with defects of different widths and depths were tested in the scanning path. The magnetic field curves were recorded for analysis. The results are as shown in Figure 11a,b. In Figure 11a, the detected five abnormal peak-to-peak signals represented five defects with different widths (0.2, 0.4, 0.6, 0.8 and 1.2 mm). For cracks with a certain width, Δ*B_xpp_* of the detected signal could reach a higher abnormal value. In Figure 11b, the detected five abnormal peak-to-peak signals represented five defects with different depths (0.5, 1.0, 1.5, 2.0 and 3.0 mm). The signal value was relatively weak for shallower cracks, but the signal increased significantly with crack depth.

When compared with the simulation results, it can be found that the trend of magnetic field change was consistent for each defect, which was consistent with the expected effect. Due to the steady-state characteristics of DC, a large number of *J_z_* induced by the crack accumulated in the depth direction, accompanied by an increase in *J_y_* in different depth layers; thus, the depth of the crack is the main influence on the magnetic signal. For the crack width, it represents the degree of the rotation of vector current density along the width, and there was some interference for the signal. From the above analysis, it follows that the *B_x_* signal can be used as an indicator for crack detection.

## 6. Analysis and Discussion

### 6.1. Characterization of Different Crack Widths

**Figure 12:**
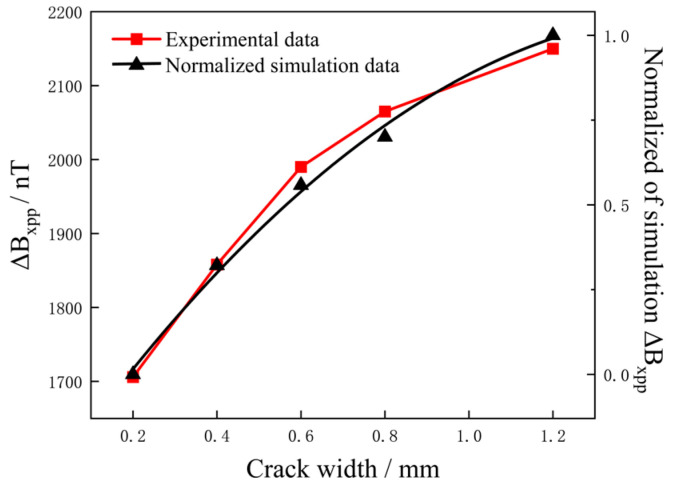
Experimental and normalized simulated results of Δ*B_xpp_* versus crack width.

The peak-to-peak value of the different widths crack were extracted, the peak-to-peak value of *B_x_*, Δ*B_xpp_*, with the crack width was as shown in Figure 12 and the normalized simulation data were essentially consistent with the test results, indicating that the finite element simulation and experiments had the same signal increase trend for cracks with different widths. The Pearson correlation coefficient was used to determine the correlation between the two groups of data [24]. The correlation was 0.9926, which confirmed the high correlation between the two data and the accuracy of the test results.

As shown in Figure 12, there was a nonlinear trend between the crack width and Δ*B_xpp_*. Under the conditions of constant crack length and depth, the increase in Δ*B_xpp_* with increasing crack width was rapid at first and then gradually slowed down. The width of the crack is an influence that increases the transverse discontinuity region and the interference area of the current, which affects the rotation degree of the density of the vector current in the width direction, leading to a non-linear enhancement of the magnetic field. The analysis revealed that the signal intensity distribution was relatively concentrated when detecting different crack widths. In the width range of 0.2 to 1.2 mm, the signal increase was 444 nT, indicating that the influence of the crack width on the detection signal was small.

### 6.2. Characterization of Different Crack Depths

**Figure 13:**
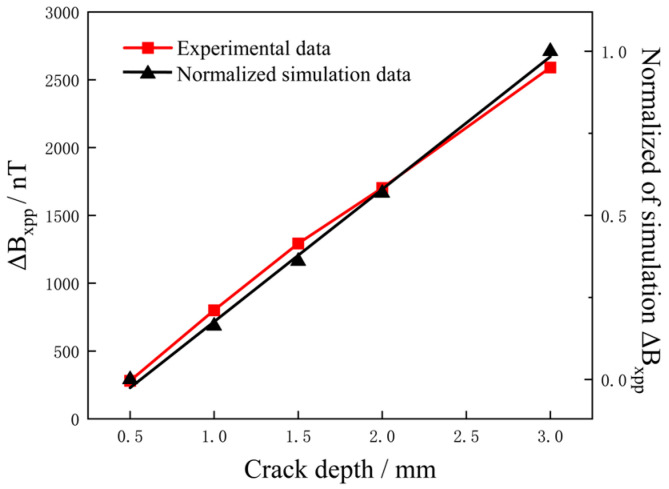
Experimental and normalized simulated results of Δ*B_xpp_* versus crack depth.

The peak-to-peak value of the different depths crack were extracted, the peak-to-peak value of *B_x_*, Δ*B_xpp_*, with the crack depths was as shown in Figure 13 and the results were as shown in Figure 13. The normalized simulation data agreed well with the test results, with both showing a strong linear relationship. The result of the correlation determination was 0.9959, indicating a high correlation between the two data.

In the trend relationship between crack depth and Δ*B_xpp_*, the influence of crack depth on Δ*B_xpp_* showed a clear linear relationship. Under the condition of constant crack length and width, Δ*B_xpp_* increased with crack depth. Compared to the influence of different crack widths, it was found that the signal intensity changes more drastically at different crack depths. In the depth range of 0.5 to 3 mm, the signal increase was 2309 nT, with an average magnetic field increase of 924 nT per 1 mm change in crack depth, indicating that the influence of crack depth on the detection signal was stronger than crack width. This phenomenon is consistent with the current perturbation theory [25]. The current was accumulated in the depth direction, and the current generating the magnetic field at the defect is the sum of the current vectors of different depth layers.

## 7. Conclusions

The above research has shown that as the width or depth of the defect increases, its signal amplitude also increases. This positive correlation was mainly due to the significant current density distortion caused by the interaction between current and cracks. Especially when the defect size is large, a stronger disturbance effect is generated around the defect, resulting in an increase in the magnetic field. This positive correlation between signal amplitude and crack size provided a theoretical basis for the quantitative evaluation of crack dimensions.

This paper presents an evaluation method for crack size interpretation based on a uniaxial method using IDC-MFM. Theoretical analysis shows that the current density *J_y_* and *J_z_* are closely related to the crack width and depth, respectively, and the obtained *B_x_* signal characteristic can explain the crack width and depth. A finite element analysis model was developed to study the effects of excitation intensity, crack width and depth on magnetic field, and a quantitative detection method using Δ*B_xpp_* as a defect was discussed.

In finite element analysis and experimental research, the magnetic field of the defect increases linearly with the excitation intensity. In the investigations range of excitation intensity, the saturated magnetic field state of the crack signal is not observed. Therefore, detection of different crack sizes can be achieved through a sensible selection of the excitation intensity. And for defects with different widths or depths, the detection technology can effectively distinguish the crack size based on the change of Δ*B_xpp_*, which is an important basis for the quantitative detection of cracks. The results provide a research direction for defect detection of non-ferrous metal components with similar material properties.

## Figures and Tables

**Table 1:** Chemical composition of the experimental material (wt%).

Brand	Si	Fe	Cu	Mn	Mg	Cr	Zn	Ti
7075 aluminum alloy	0.4	0.5	1.2–2.0	0.3	2.1–2.9	0.18–0.28	5.1–6.1	0.2

**Table 2:** Physical properties of the experimental material.

Brand	Conductivity [m/Ω·mm^2^]	Coefficient of Thermal Expansion [K^−1^·10^−6^]	Thermal Conductivity [W/m·K]
7075 aluminum alloy	19–23	23.4	196

## Data Availability

The data presented in this study are available on request from the corresponding author.
